# Motor Innervation of the Trapezius by Cervical Sensitive Nerves: An Intraoperative Neuromonitoring Study and Postoperative Functional Outcomes

**DOI:** 10.3390/healthcare13182297

**Published:** 2025-09-13

**Authors:** Enver Can Öncül, Ersoy Doğan, Aslı Çakir Çetin, Aybüke Cansu Kalkan, Seher Özyürek, Arzu Genç, Ahmet Ömer İkiz

**Affiliations:** 1 Department of Otorhinolaryngology, Dokuz Eylul University, Izmir 35000, Turkey; 2 Faculty of Physical Therapy and Rehabilitation, Dokuz Eylul University, Izmir 35000, Turkeyseher.ozyurek@deu.edu.tr (S.Ö.);

**Keywords:** cancer of head and neck, neck dissection, monitoring, intraoperative, cervical plexus

## Abstract

Background/Objectives: We investigated whether cervical sensitive nerves (CSN) provide motor input to the trapezius muscle and how this relates to short-term functional outcomes after neck dissection. Methods: A total of 22 neck dissections were performed in 17 patients; the SAN was preserved. CSN roots (C2–C4) were stimulated intraoperatively using IONM. Shoulder and neck function were evaluated preoperatively and at 3 months in 15 operated necks using goniometry, an IMU-based motion analysis system (iSen), trapezius isometric strength, the Modified Constant–Murley Score (MCMS), and the Shoulder Pain and Disability Index (SPADI). Results: CSN-evoked trapezius responses were detected in 10/22 (45.5%) dissections (C2: 6/22, 27.2%; C3: 4/22, 18.2%; C4: 0/22). Postoperatively, neck extension/rotation and shoulder abduction/external rotation decreased significantly within groups; upper and middle trapezius strength and MCMS also declined. Shoulder flexion loss was smaller when CSN motor participation was present. Median shoulder flexion (goniometry) changed from 162°→140° in CSN(+) vs. 170°→131° in CSN(−) (between-group *p* = 0.024). With iSen, shoulder flexion changed 120°→116° in CSN(+) vs. 122°→97° in CSN(−) (*p* = 0.033). Conclusions: Approximately half of the neck dissections exhibited CSN-related motor responses. Short-term shoulder flexion was better preserved when CSN motor participation was present, suggesting that documenting CSN motor input intraoperatively may inform early rehabilitation planning.

## 1. Introduction

Cancers of the oral cavity and head and neck represent some of the most prevalent types of cancer worldwide and are a significant cause of morbidity [1]. Historically, during the initial management of these malignancies, radical neck dissection (RND) was the standard procedure. One of the well-known consequences of radical neck dissection is spinal accessory nerve (SAN) resection, which leads to denervation and atrophy of the trapezius muscle, shoulder drop, decreased shoulder muscle strength, and limited active range of motion in the shoulder joint, ultimately contributing to frozen shoulder syndrome [2]. These challenges have spurred the development of other surgical procedures that aim to preserve the SAN without compromising oncological principles, such as modified radical and selective neck dissections.

However, the results of nerve resection and nerve-sparing procedures vary. Despite preservation of the SAN, shoulder dysfunction may occur in a significant proportion of cases [3,4,5,6,7,8]. This result suggests variations in the innervation of the trapezius muscle, which plays an important role in shoulder function. Although anatomical and histological studies have investigated the contributions of the C2, C3, and C4 branches of the cervical plexus to the SAN and trapezius muscle, no study has examined the effects of these variations on neck-shoulder function [2,3,4,9,10,11,12,13,14].

We aimed to investigate the motor participation of cervical sensitive nerves (CSN) in the innervation of the trapezius muscle using intraoperative neuromonitoring (IONM) and to compare the groups with and without motor function in terms of physical therapy scores.

It is known that these nerves are resected in some surgeries due to their lack of significance. Our goal is to examine whether these nerves have motor function and, if so, their impact on neck and shoulder movements. This will allow us to tailor postoperative rehabilitation programs based on the function of these nerves.

## 2. Materials and Methods

The study was a prospective observational investigation conducted by the ethical guidelines established by the Dokuz Eylul University Research Ethics Committee (2022/20-02). The study was conducted in accordance with the guidelines of the 1975 Helsinki Declaration, as revised in 2013. Data collection was conducted between August 2022 and August 2023 at the Dokuz Eylul University Department of Otorhinolaryngology and the Dokuz Eylul University Faculty of Physical Therapy and Rehabilitation. An informed consent form was obtained from all patients.

Exclusion Criteria:

- Current shoulder–neck pain.
- Limited shoulder/neck mobility within 3 months before surgery.
- History of surgery or trauma in the shoulder/neck region.
- Loss of SAN or CSN integrity due to tumor invasion before dissection.

Inclusion Criteria:

- Availability of standardized preoperative and 3-month postoperative physiotherapy assessments.
- Preservation of the SAN during dissection (levels II and V explored).
- Patients with head and neck squamous cell carcinoma undergoing neck dissection by the same surgical team.

Twenty-two neck dissections performed on 17 patients due to head and neck squamous cell carcinoma, operated on by the same expert surgical team, were included in this study. The dissections reveal the SAN and CSN from the skull base to the sternocleidomastoid muscle (SCM) entry point. During the neck dissection procedure, the SAN was preserved by dissecting the trapezius muscle entry at level V in addition to level II. Four neck dissections were modified radical neck dissections (MRNDs), and 18 were selective neck dissections (SNDs). Patients whose SAN or CSN integrity could not be preserved due to tumor invasion before proceeding to the neck dissection phase, patients with a history of surgery or trauma in the shoulder or neck region, patients with limited shoulder or neck movement within three months before surgery, and patients with shoulder-neck pain were excluded from the study. Physical therapy evaluations, an important part of the study, were performed for 15 of the 22 neck dissections.

### 2.1. Intraoperative Evaluation

All patients underwent intraoperative neuromonitoring (IONM) for SAN and CSN during surgery. The device used was the “NIM-Response 3.0 Nerve Integrity Monitoring System (Medtronic, Jacksonville, FL, USA)”.

**Figure 1:**
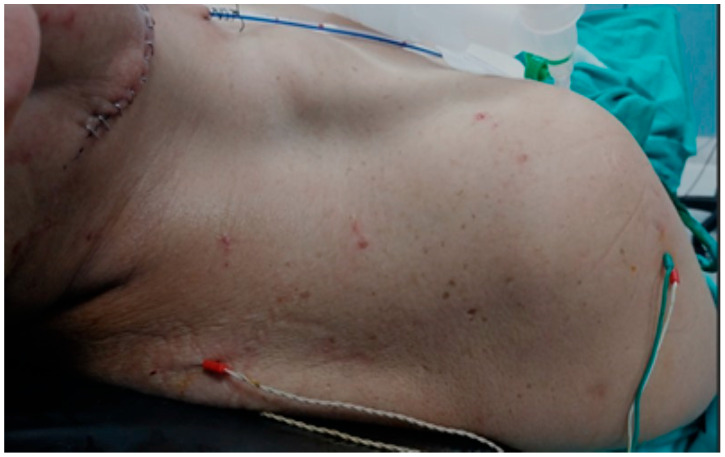
Placement of Electrodes (Intraoperative neuromonitoring involves stimulating the nerve during surgery and observing the EMG responses in the muscle. In this procedure, a ground electrode is placed in the deltoid muscle (shoulder), while other electrodes are placed in the upper, middle, and lower fibers of the trapezius muscle).

A double-needle stainless steel electrode was placed five cm lateral to the C7 vertebra processus spinosus for the descending fibers of the trapezius muscle. A double-needle stainless steel electrode was placed in the middle of the distance between the spina scapula and the processus spinosus of the vertebra at the same level for transverse fibers. A double-needle stainless steel electrode was placed three cm lateral to the T7 vertebra processus spinosus at a right angle for the ascending fibers. Grounding electrodes were placed on the side of the deltoid muscle, where neck dissection was performed [4] (Figure 1). A monopolar nerve stimulator was used for stimulation during the operation.

0.3 mg/kg rocuronium bromide was preferred as a muscle relaxant. It was administered only by anesthesiologists during anesthesia induction, and patients were not given additional doses that could affect IONM results.

**Figure 2:**
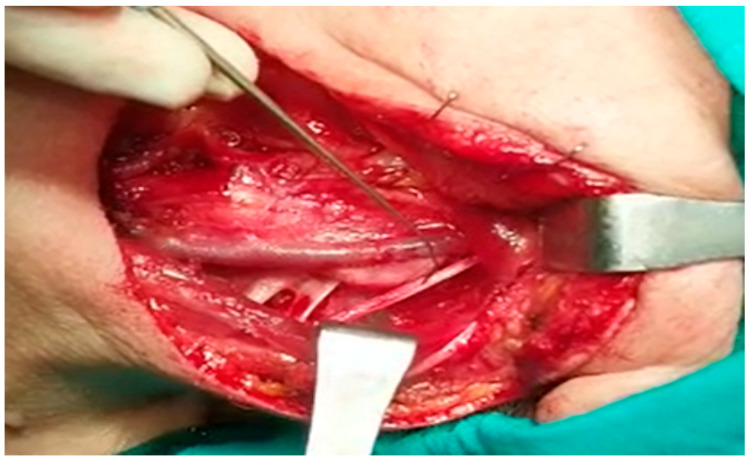
Stimulation of spinal accessory nerve during surgery: The spinal accessory nerve is the main nerve of the trapezius muscle. During neck dissection, the spinal accessory nerve is stimulated to check whether there is contraction in the trapezius muscle and whether the system is functioning.

**Figure 3:**
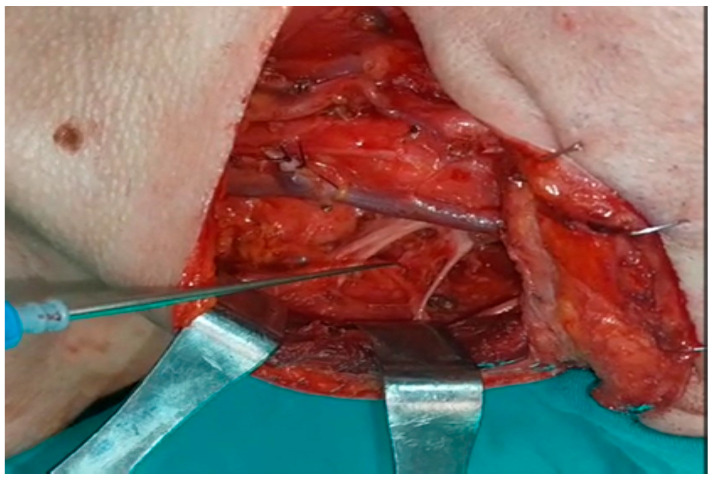
Stimulation of the cervical sensitive nerves during surgery: The main nerve of the trapezius muscle is the spinal accessory nerve. However, the role of the cervical sensory nerves in innervating this muscle is unknown. Here, the cervical sensory nerves are stimulated to determine whether the trapezius muscle contracts and its amplitude responses.

When SAN was first recognized and neck dissection was completed, the SCM was stimulated just proximal to the entry site for control purposes, and the amplitudes in the ascending, transverse, and descending fibers of the trapezius muscle were recorded separately. During dissection, the amplitudes in the ascending, transverse, and descending fibers of the trapezius muscle were recorded independently by following the n.auricularis magnus at the base or the n.supraclavicularis proximally if seen, finding the C2, C3, and C4 primary roots deep in the SCM and stimulating them separately (Figure 2 and Figure 3).

### 2.2. Determination of Amplitude

The nerves were stimulated six times, one by one, at intervals of 1 mA, and the amplitude values of the EMG waves formed in the trapezius muscle were recorded in millivolts (mV). The average of these values was taken. At the same time, it was evaluated whether there was a pulse in the trapezius muscle during stimulation and the only cases that demonstrated stimulation were included in the study. These data were recorded in the data recording form. When the initial and final SAN amplitudes were compared, an amplitude loss of 72% or more was considered indicative of SAN impairment [15,16].

### 2.3. Physical Therapy Evaluation

All assessments were conducted by the same team of three experienced physiotherapists, both before surgery and at the 3-month follow-up. No physiatrist was involved in the evaluation process.

### 2.4. Range of Motion Measures

A goniometer and motion capture system evaluated the active ranges of motion (RoM) of the shoulder (flexion, extension, abduction, internal and external rotation) and neck (flexion, extension, lateral flexion, and rotation).

#### 2.4.1. Goniometric Measures

A manual extendable-arm steel goniometer (Lafayette Gollehon, Model 011135, Lafayette Instrument Company, Loughborough, Leics., UK) was used for goniometric measurements. Shoulder measures were performed in the supine position for flexion, abduction, and internal and external rotation, as well as in the prone position for extension. Neck RoM was assessed seated on a chair with a backrest supporting the thoracic and lumbar spine. The assessment methods of each measurement are described below [17].

*Shoulder flexion and extension*: The goniometer pivot was positioned on the greater tubercle of the humerus. The proximal arm of the goniometer was parallel to the midaxillary line of the thorax, while the distal arm was placed on the lateral midline of the humerus. To evaluate shoulder flexion RoM, the participant was asked to lift the humerus from the examination table by holding the extremity in 0° abduction and adduction and to place the hand over the head. To assess shoulder extension RoM, the participants were asked to lift the humerus from the examination table by holding the extremity in 0° abduction and adduction.

*Shoulder abduction*: The goniometer pivot was positioned on the acromion process of the scapula’s anterior surface. The proximal arm of the goniometer was parallel to the midline of the sternum’s anterior surface, while the distal arm was placed on the anterior midline of the humerus. To measure shoulder abduction RoM, the participant was asked to open the arm with the humerus away from the trunk.

*Shoulder internal and external rotations*: The patient’s arm was placed in 90° abduction, 0° pronation, and supination, and a towel was placed under the humerus. The humerus was positioned on the examination table, while the elbow was off. The goniometer pivot was positioned on the olecranon. The proximal arm of the goniometer was perpendicular to the floor, while the distal arm was placed on the ulna, taking the ulnar styloid process as a reference. While the participant was asked to move the forearm forward and to approach the hand towards the floor for shoulder internal rotation, the participant was asked to move the forearm backward and to approach the hand towards the floor for shoulder external rotation.

*Neck flexion and extension:* The goniometer pivot was positioned on the external auditory meatus. The proximal arm of the goniometer was placed parallel to the floor, while the distal arm was positioned to follow the base of the nostrils. To assess neck flexion RoM, the participants were asked to bring their chin towards their sternum until resistance to the movement was felt. The participant was asked to look up to measure RoM in neck extension while moving the head backward.

*Neck lateral flexion:* The goniometer pivot was positioned on the C7 spinous process. The proximal arm of the goniometer was perpendicular to the floor, taking the spinous processes of the thoracic spine as a reference. In contrast, the distal arm was positioned to coordinate with the head midline using the occipital prominence as a reference. To evaluate neck lateral flexion RoM, the participant was asked to touch the ear to the shoulder without rotating or extending the head.

*Neck rotation:* The goniometer pivot was placed on the center of the cranium. The proximal arm of the goniometer was parallel to the imaginary line between the right and left acromial processes, while the distal arm aligned with the nasal tip. To measure neck rotation RoM, the participants were asked to turn their heads by tilting their chin towards their shoulders without any trunk movement.

In the goniometric evaluation, all measurements were recorded as degrees. Three measurements were taken for each movement, and the average of these measurements was used for statistical analysis.

#### 2.4.2. Motion Capture System Evaluation

**Figure 4:**
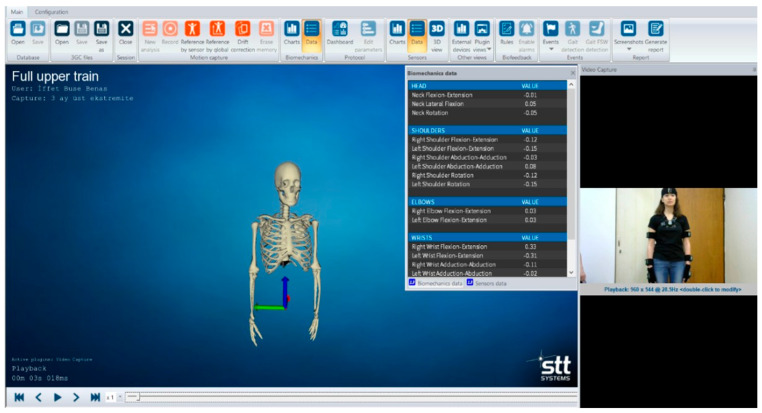
iSen system (STT systems Company, Spain) (Thanks to this system, the patient’s joint range of motion is measured through a computer program).

In addition to goniometric measurements, active RoM was evaluated using an inertial measurement unit (IMU) system (iSen, STT Systems Company, San Sebastián, Spain). The system is a device that enables biomechanical analysis, biofeedback, and reporting opportunities, performs motion analysis quickly and easily, and provides real-time recording of human movements. The iSen enables three-dimensional visualization of the skeleton using wearable inertial sensors, including accelerometers, magnetometers, and gyroscopes, by the motion analysis protocol [18]. The transfer rate of data collected from sensors is 25–400 Hz, depending on the number of sensors. The data is transferred to a computer using the system’s software via a wireless Wi-Fi connection [19]. The data were processed using the software iSen 3.08 for the IMU system (Figure 4).

The “Full Upper Train” protocol, one of the standard evaluation protocols in the iSen, was used to assess active RoM in the shoulder and neck. A total of eight inertial sensors, including forehead, sternum, right/left upper arm, right/left forearm, and right/left hand, were placed on the participant’s body using the velcro straps. Each movement was demonstrated to participants on the evaluator’s body before and during the recording. In the upright position, RoMs for the shoulder, including flexion, extension, abduction, and adduction, and for the neck, flexion, extension, lateral flexion, and rotation were assessed. For shoulder flexion, the participant was asked to raise the arm as high as possible with the elbow in full extension. For shoulder extension, the patient was asked to move the arm backward without abduction or adduction with the elbow in full extension. The participant was asked to raise the arm sideways with the palm facing forward and the elbow full extension for shoulder abduction. For shoulder adduction, the patient asked to move the arm back down and bring it towards the trunk. The participant was asked to bring their chin towards their sternum and move their head forward for neck flexion. The patient was asked to raise the chin and move the head backward for neck extension. For neck lateral flexion, the participant was asked to bring the ear toward the same side of the shoulder without head rotation. For the neck rotation, the patient asked to turn the head without lateral bending.

The patient was asked to perform shoulder and neck RoM in the maximum range without compensatory trunk movements. Each movement was assessed three times, and the values were recorded in degrees. The average of these measurements was used for statistical analysis.

#### 2.4.3. Muscle Strength

The upper, middle, and lower trapezius isometric strength was assessed using a handheld dynamometer (Lafayette Instrument, Lafayette, IN, USA). During the assessment process, the participant was asked to apply maximal strength against the resistance of the dynamometer [20].

The upper trapezius muscle was evaluated with the participant seated without back support. The participant’s head was positioned in lateral flexion towards the side being assessed, with rotation towards the opposite side and extension. The dynamometer was placed above the shoulder, and one hand applied resistance to the head. In contrast, downward resistance was applied to the shoulder with the dynamometer on the other hand [21].

The middle trapezius muscle was assessed in the prone position. The participant’s arm was positioned in horizontal abduction, full extension, and external rotation. The dynamometer was placed at 1/3 of the distance from the acromion to the spinal root. The resistance was applied in the anterolateral direction [20].

The lower trapezius muscle was evaluated in the prone position. The participant’s arm was positioned in 140° flexion and external rotation. The dynamometer was placed on the spine of the scapula, approximately 1/3 of the length from the acromion to the spinal root. The resistance was applied in the superior and anterolateral directions [20].

Three strength measurements were performed for each part of the trapezius muscle, and the average value of these measures was recorded as kilograms. While a rest period of 30 s was allowed between the measurements of the same part of the trapezius muscle, a rest period of 60 s was allowed between the measurements of the different parts of the trapezius muscle [20].

#### 2.4.4. Muscle Endurance

The static scapular muscular endurance test was used to evaluate muscle endurance. The test examines the endurance of the trapezius and serratus anterior muscles. The patient was standing, facing the wall, with shoulders and elbows flexed at 90°. A ruler of two length-adjustable interlocking metal blocks was placed between the participant’s elbows in the neutral scapular position. The dynamometer (Scale and Tape Measure, Design No 990205) was placed between the participant’s hands. The participant was asked to reach a load of 1 kg against the dynamometer with external rotation and to maintain the load. The test stopped if the participant could not maintain the load, dropped the ruler, or was unable to continue in the 90° flexed position of the shoulders and elbows [22,23]. The test was repeated twice, with a 5 min rest period provided between trials. The time was recorded with a stopwatch in seconds. The maximum score was used for statistical analysis.

#### 2.4.5. Shoulder Functionality

To evaluate shoulder function, the Modified Constant–Murley Score (MCMS) and Shoulder Pain and Disability Index (SPADI) were used.

The MCSM is a standard scale for evaluating shoulder disability. The Turkish version of the MCSM is a reliable and valid instrument. It consists of four subscales: pain, activities of daily living, strength, and range of motion. The total score is evaluated on a scale of 100 points, comprising 35 points for subjective parameters, including pain and activities of daily living, and 65 points for objective parameters, including strength and range of motion. A higher score indicates better shoulder function [24].

The SPADI is a self-report questionnaire developed to assess shoulder pain and disability. The Turkish version of SPADI is reliable and valid. It consists of 13 items, including five items for the pain and eight for the disability. All items of these two subscales are investigated with visual analog scales as “0 points: no pain and difficulty” and “10 points: the most intense pain imaginable and so difficult as to require assistance”. The “(Total pain score/50) × 100” formula is used for the total pain score, while the “(Total disability score/80) × 100” formula is used for the total disability score. The average total pain and disability scores were calculated for the total SPADI score. A higher score indicates worse pain and disability [25].

#### 2.4.6. Statistical Analysis

In the data analysis, IBM Corp. Released 2017 IBM SPSS Statistics for Windows, Version 25.0. Armonk, NY, USA: The IBM Corp program was utilized. The suitability of the variables to normal distribution was examined using histogram graphics and the Shapiro–Wilk test. Mean, standard deviation, median, and minimum-maximum values were used when presenting descriptive analyses. Mann–Whitney U Test was used to evaluate non-parametric variables between groups. The change in measured values was examined within the group with the Wilcoxon Test and between the groups with the Repeated Measures Analysis. Cases where the *p*-value was below 0.05 were considered statistically significant results. At a 95% confidence level with a 5% worst-case error, including the duration of the study, the inclusion of at least 15 neck dissections is sufficient for statistical evaluation. Calculations were made using the Openepi program(Version 3.01).

## 3. Results

Three neck dissections had a decrease in SAN amplitude. Of the three patients with decreased SAN amplitude, two were operated for laryngeal malignancy and one for oral cavity malignancy. No CSN motor participation was detected in any patient with a loss of SAN amplitude.

**Figure 5:**
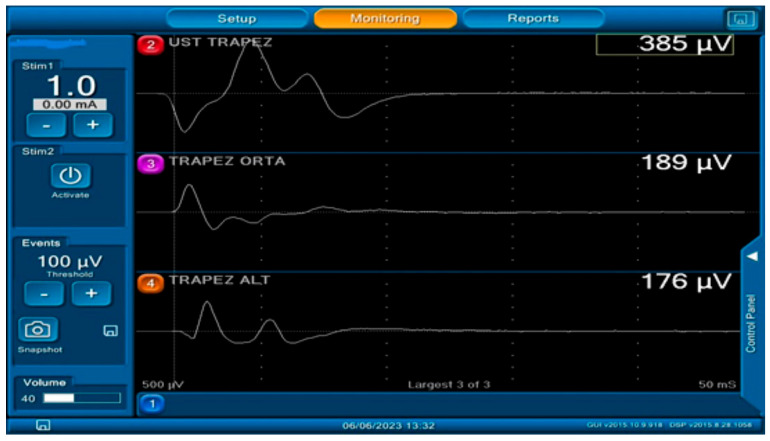
Intraoperative neuromonitorisation responses when stimulation is given to the left-sided C2 nerve root (In the literature, the main and only nerve of the trapezius muscle is known as the spinal accessory nerve. Here, stimulation of one of the branches of the cervical sensory nerve produces a motor response in the trapezius muscle).

Upon evaluation of CSN participation, according to IONM responses, six patients had motor innervation of the C2 root of the trapezius muscle, while four different patients had motor innervation of the C3 root (Figure 5). No motor innervation from the C4 root was observed in any patient. Table 1 shows the distribution responses of motor responses generated by IONM and trapezius fibers of CSN.

The physical therapy scores of individuals with and without CSN motor participation were examined for changes within the group preoperatively and at the 3-month postoperative follow-up, as well as between the groups, in the third month. When joint ranges were evaluated within the CSN motor participation group itself, neck extension and rotation, shoulder abduction, and external rotation ranges demonstrated a statistically significant reduction in postoperative goniometric and iSEN device measurements.

When the group without CSN motor participation was evaluated within itself, the ranges of neck extension and rotation, shoulder flexion, abduction, and external rotation demonstrated a statistically significant reduction.

After surgery, the isometric strength of the upper and middle trapezius fibers demonstrated a statistically significant reduction in both groups, while the patients’ MCMSs decreased.

A notable finding was that when the groups with and without CSN motor participation were compared, the shoulder flexion score in the measurements made with goniometry and the iSEN device demonstrated a statistically significant reduction in the group without motor participation compared to patients with confirmed CSN motor participation (Table 2).

## 4. Discussion

Consistent with previous clinical studies, postoperative deterioration in shoulder function was observed regardless of SAN preservation. However, our results indicate that patients with detectable CSN motor input demonstrated better preservation of shoulder flexion. This finding highlights the potential compensatory role of CSNs in supporting trapezius activity when SAN function is compromised. Recent systematic reviews emphasize that structured physiotherapy, particularly progressive resistance training and scapular stabilization exercises, can improve shoulder flexion and abduction after neck dissection [26]. Thus, our observation of preserved flexion in CSN(+) patients may have direct rehabilitative implications.

Neck dissection is a surgical approach used in the staging and treatment of cervical lymph node metastases in patients with head and neck cancer. Radical neck dissection (RND) was the primary neck dissection technique performed for many years. Sacrificing the spinal accessory nerve (SAN) in this procedure may cause a deficiency in shoulder functions. Excision of the SAN leads to denervation and atrophy of the trapezius muscle, the emergence of shoulder drop, decreased shoulder muscle strength, and limited active range of motion in the shoulder joint, resulting in “frozen shoulder syndrome” [5,6,7,26,27,28,29]. Since the introduction of functional neck dissection techniques, various modifications have been made to mitigate the adverse effects of the RND technique and ensure adequate oncological effectiveness. In this way, it aimed to increase patients’ quality of life after neck dissection and prevent permanent sequelae [30,31]. To mitigate these postoperative complications, it has become essential to understand the variations in trapezius muscle innervation. One of the most critical variations may be the contributions of the cervical plexus’ C2, C3, and C4 branches to the SAN and trapezius muscle.

Moreover, neurophysiological and anatomical studies increasingly support the view that motor contributions from C2–C4 can supplement SAN innervation, especially following partial nerve injury [32]. These findings are consistent with our intraoperative results, where stimulation of C2 and C3 roots elicited measurable trapezius responses. Preservation of these branches during surgery could therefore mitigate functional decline and may be particularly important in patients at higher risk of SAN impairment.

Birinci et al. conducted a prospective study preserving the SAN. In this study, statistically significant deteriorations in shoulder functions were detected in all patients in both Constant–Murley Shoulder Scores and the Gröningen Activity Limitation Scale in the first and second postoperative months, compared to the measurements made in the preoperative period [16]. Consistent with this study, Modified Constant–Murley Score (MCMS) scores demonstrated a statistically significant reduction in both groups with and without cervical sensitive nerve (CSN) motor participation. Interestingly, when we compared these two studies, we found that the number of patients with SAN amplitude loss was lower in our study. Of the three patients with decreased SAN amplitude, two were operated on for laryngeal malignancy and one for oral cavity malignancy. In neck dissections of oral cavity malignancies, where dissection of the cervical 2B region is performed and more manipulation is performed on the SAN, SAN amplitude can decrease. The difference between the two studies conducted in the same clinic may be due to the significant effect of the current trend on the indication of cervical 2B region dissection. This indication has narrowed over the years. Additionally, operation times have shortened due to increased surgical experience. At the same time, there may be differences in these rates depending on whether the neck dissections are performed for therapeutic or elective purposes.

Upon examining the literature, it becomes apparent that the findings of studies on the motor contribution of CSNs to trapezius muscle innervation are contradictory. Soo et al., in their research on 23 cadavers, found that the C2 nerve root supplied branches to the trapezius muscle in 65.5% of the dissections, C3 in 31%, and C4 in 3% [33]. Kierner et al., in 44 dissections they performed on cadavers, detected a branch from the CSN to the trapezius muscle in 9% of the dissections, while they detected two branches in 61% and three branches in 30% [33]. Gavid et al., in their study, performed 23 anatomical dissections on 12 cadavers. They detected anastomoses between the C2 nerve root and the SAN in 78% of the dissections, the C3 nerve root in 48%, and the C4 nerve root in 52% [2]. However, CSN branches were not examined in terms of motor or proprioceptive branch content in these studies.

Gavid et al.’s study, which used intraoperative neuromonitoring (IONM) on 25 modified radical neck dissections (MRNDs), observed visible contraction in the trapezius muscle with stimulation of the C2 nerve roots in 7%, C3 in 20%, and C4 in 20% of the patients. While the C2 nerve root stimulates all trapezius muscle fibers in 7% of patients, the C3 root has descending fibers in 20% of patients and transverse and ascending fibers in 12%. The C4 root stimulated the descending and transverse fibers in 16% of the patients, while it stimulated the ascending fibers in 20%. As a result, they found that the CSN had motor functions on the trapezius muscle in 32% of the patients. They reported that these branches should be protected to prevent shoulder movement dysfunction [2]. In contrast to the findings of this study, Kierner et al. did not stimulate the CSN to cause any contraction in their research using IONM on 17 MRND in 14 patients [14].

Kim et al.’s study using IONM on 24 neck dissections found that C2 nerve root stimulation stimulated the descending fibers in 8% of the patients and the transverse fibers in 4% of the patients but did not stimulate the ascending fibers in any of them. When the C3 nerve root was stimulated, descending fibers contracted in 32% of patients, transverse fibers contracted in 46%, and ascending fibers contracted in 38% of patients. When the C4 nerve root was stimulated, it was observed that the descending fibers contracted in 64% of the patients, the transverse fibers contracted in 83% of the patients, and the ascending fibers contracted in 71% of the patients [4]. Pu et al., in their study on 34 patients, found that C2 nerve roots transmitted motor impulses to the trapezius muscle via the SAN, while C3 and C4 nerve roots transmitted them independently. Considering the IONM and histological findings, it was observed that the C2 nerve root in 44% of the patients, C3 in 64%, and C4 in 61% contained motor fibers innervating the motor trapezius muscle [3].

Svenberg et al.’s study of 18 neck dissections detected CSN motor branches in 39% of the patients at a level comparable to the SAN, providing equal stimulation to all three functional parts of the trapezius muscle. They also stated that independent of the normal physiological relationship between the SAN and the CSN, the motor contribution from the CSN is essential in reinnervation after SAN axonal injury [9]. In our study, amplitude in the trapezius muscle was obtained when the CSN was stimulated in 10 of 22 neck dissections (45.5%) with IONM. Six (27.2%) originated from the C2 root, while four different (18.2%) were derived from the C3 root. No response was obtained from the C4 root in any case. While a response was obtained in the descending and transverse fibers of the trapezius muscle in six (27.2%) patients in whom the C2 root was stimulated, an amplitude was obtained in the ascending fibers of the trapezius muscle in three of the same patients (13.6%). When the C3 root was stimulated, a response was obtained in the descending and transverse fibers of the trapezius muscle in four (18.2%) operations.

In comparison, a response was obtained in the ascending fibers of the trapezius muscle in three of the exact dissections (13.6%). When the CSN was stimulated, an amplitude was obtained in the descending and transverse fibers of the trapezius muscle in 10 (45.5%) neck dissections. A response was obtained in the trapezius ascending fibers in six of these 10 neck dissections (27.2%). Upon examining the literature, it becomes apparent that the CSN motor participation rates differ. These nerves can be stimulated at the end of the neck dissection. They can be seen at the dissection base. As a result, they may enter neuropraxia due to the manipulations made during the operation.

When the literature is examined, studies evaluate physical therapy scores for the neck and shoulders after neck dissection. However, no study compares the neck and shoulder functions of patients with and without motor innervation of the CSN.

Popovski et al. found the rate of frozen shoulder syndrome after RND to be 46.7%. After selective neck dissection (SND), they observed a decrease in shoulder functions in 42.5% of the patients compared to preoperative values [30]. Gane et al., although they could not detect a connection between the type of neck dissection and decreased shoulder motor function in their study, showed that shoulder motor function decreased in the neck dissection group compared to the healthy control group [5].

Finally, contemporary rehabilitation-focused publications recommend early referral to specialist physiatrists and physiotherapists to implement individualized postoperative exercise programs [34,35]. Although no standardized physiotherapy protocol was applied in our study, the identification of CSN motor participation may serve as a practical indicator for tailoring early rehabilitation. In particular, patients without CSN participation may require more intensive strategies to preserve shoulder flexion and abduction, while patients with confirmed CSN input might benefit from a more gradual approach.

Özyürek et al. found that patients had decreased trapezius and serratus anterior muscle strength and scapular endurance in the third month after the operation. They also found a medium-strong clinical correlation between MCMS and other shoulder muscle strengths, except upper trapezius muscle strength and scapular endurance change [23,36,37]. Kalkan et al., in a study conducted on 12 patients who underwent 17 neck dissections, found a significant decrease in the thickness of the trapezius transverse and ascending fibers after neck dissection. However, they did not detect any change in scapular endurance, unlike a previous study [38]. Özyürek et al. found a correlation in another study between trapezoidal transverse fiber thickness, measured by ultrasonographic imaging, and shoulder abduction and scapular endurance after neck dissection. The exact correlation was not observed in the trapezium ascending and descending fibers. For this reason, they concluded that exercises for the transverse trapezius fibers after neck dissection would reduce postoperative shoulder movement dysfunction [39]. In our study, a decrease in muscle strength was detected in all fibers of the trapezius; However, the reduction in the isometric strength of the upper and middle trapezius fibers was found to be statistically significant. (with and without participation, respectively, upper trapezius *p* = 0.18 and *p* = 0.12, middle trapezius *p* = 0.28 and *p* = 0.12) Although a reduction in scapular endurance was detected, this decrease was not statistically significant (with and without participation, respectively, *p* = 0.176 and *p* = 0.574). In the group with motor participation, better shoulder flexion scores were obtained after the operation than in the group without motor participation (*p* = 0.024). This is the most striking finding of our study from our perspective.

However, these studies have limitations. More accurate generalizations can be made with larger case series. In addition, physical therapy scores may vary depending on the patient’s age, gender, dominant hand use, sports capacity, smoking and alcohol use, and concomitant diseases. Additionally, the physical therapy scores obtained in this study were collected three months after the surgery. We believe that longer follow-ups and additional evaluations are necessary to obtain more accurate data.

### Limitations

Our study has several limitations that should be acknowledged.

The study included only 22 dissections in 17 patients, with physiotherapy evaluations performed in 15 operated necks. Although a sample size calculation was performed with OpenEpi, no post hoc power analysis was conducted.

The study design was prospective but lacked an external healthy control group, and there was no randomization of patients into (+CSN/−CSN) groups. The analysis was anatomical in nature and uncontrolled, which may limit causal inferences.

Potential confounding variables such as age, sex, handedness, smoking and alcohol use, and comorbidities were not adjusted using multivariate models, which may have influenced the results.

All patients were operated on by the same surgical team, which ensures standardization but may also represent a selection bias, limiting the generalizability of the findings.

The follow-up period was limited to three months. Functional recovery after oncological surgery and neuromuscular rehabilitation may become more evident at 6–12 months, and our results therefore represent only short-term outcomes.

All assessments were performed by the same physiotherapist team to ensure consistency. However, inter- and intra-rater reliability was not separately calculated.

Statistical analyses were performed using Wilcoxon and repeated measures analysis. However, no Bonferroni or Holm corrections for multiple comparisons were applied, and confidence intervals and effect sizes were not reported, increasing the risk of type I error.

The study involved repeated measurements, but no explicit control for learning or fatigue effects was described, which may have introduced bias.

Finally, although ethical approval and informed consent were reported, the study protocol was not registered in a public clinical trial registry (e.g., ClinicalTrials.gov), which represents an additional limitation.

## 5. Conclusions

Neck dissections have a negative impact on physical therapy scores in the short term. Knowing the innervation variations in the trapezius muscle may give us an advantage during surgery. It is evident that CSN motor participation rates, one of the variations examined in the literature, vary. According to our study, shoulder flexion scores were found to be higher in the short term in the group with motor participation. However, studies with longer-term follow-up and evaluation of patients are needed. In this way, rehabilitation programs following neck dissection can be customized based on the presence or absence of CSN motor participation in the trapezius muscle.

## Figures and Tables

**Table 1:** Distribution of motor contributions detected by intraoperative neuromonitor in the upper-middle-lower fibers of the trapezius muscle of the cervical sensitive nerve roots within the neck dissection (total n: 22).

	C2 Trapezius Total n (%)			C3 Trapezius Total n (%)			C4 Trapezius Total n (%)			CSN * Total n (%)		
Fibers	Upper n(%)	Middle n(%)	Lower n(%)	Upper n(%)	Middle n(%)	Lower n(%)	Upper n(%)	Middle n(%)	Lower n(%)	Upper n(%)	Middle n(%)	Lower n(%)
Response	6 (27.2)			4 (18.2)			0 (0)			10 (45.5)		
	6 (27.2)	6 (27.2)	3 (13.6)	4 (18.2)	4 (18.2)	3 (13.6)	0(0)	0(0)	0(0)	10 (45.5)	10 (45.5)	6 (27.2)

* CSN: cervical sensitive nerves.

**Table 2:** Within- and between-group distributions of functional outcomes in patients with and without CSN motor participation. Range of motion (ROM) values are expressed in degrees (°), strength in kilograms (kg), and endurance in seconds (s). P1/P2: Wilcoxon signed-rank tests (within-group); P3: repeated-measures analysis (between groups).

	CSN * Attendance		P1 [Within CSN (+)]	P2 [Within CSN (−)]	P3: Between Groups
	Yes	No			
	Median (Min-Max)	Median (Min-Max)			
Neck flexion before surgery	55 (34–76)	55 (36–74)	0.063	0.105	0.480
Neck flexion after surgery	50 (30–71)	50 (31–75)			
Neck extension before surgery	47 (30–64)	48 (31–68)	**0.018**	**0.012**	0.264
Neck extension after surgery	32 (12–43)	32 (12–42)			
Neck lateral flexion before surgery	33 (21–58)	32 (23–45)	0.063	0.260	0.685
Neck lateral flexion after surgery	25 (15–42)	27 (12–42)			
Neck rotation before surgery	78 (66–88)	79 (65–88)	**0.018**	**0.012**	0.395
Neck rotation after surgery	58 (42–76)	54 (45–70)			
Shoulder flexion before surgery	162 (148–179)	170 (155–180)	0.553	**0.018**	**0.024**
Shoulder flexion after surgery	140 (136–169)	131 (125–152)			
Shoulder extension before surgery	52 (39–75)	46 (30–68)	0.499	0.236	0.547
Shoulder extension before surgery	42 (30–62)	38 (30–69)			
Shoulder abduction before surgery	170 (146–180)	173 (164–180)	**0.018**	**0.017**	0.297
Shoulder abduction after surgery	150 (117–175)	152 (119–178)			
Shoulder internal rotation before surgery	85 (71–90)	84 (70–90)	0.157	0.109	0.997
Shoulder internal rotation after surgery	79 (59–90)	79 (61–90)			
Shoulder external rotation before surgery	86 (72–90)	86 (74–90)	**0.018**	**0.017**	0.080
Shoulder external rotation after surgery	75 (40–90)	73 (42–90)			
iSEN shoulder flexion before surgery	120 (83–143)	122 (83–136)	0.114	**0.017**	**0.033**
iSEN shoulder flexion after surgery	116 (83–142)	97 (79–120)			
iSEN shoulder extension before surgery	38 (3–54)	42 (6–64)	0.866	0.674	0.861
iSEN shoulder extension after surgery	37 (14–49)	41 (17–58)			
iSEN shoulder abduction before surgery	155 (115–180)	150 (78–180)	**0.017**	**0.018**	0.080
iSEN shoulder abduction after surgery	131 (63–178)	126 (44–169)			
iSEN shoulder adduction before surgery	−1 (−9–14)	−1 (−5–12)	0.310	0.778	0.281
iSEN shoulder adduction after surgery	−3 (−12–14)	−2 (−10–13)			
iSEN neck flexion before surgery	48 (8–72)	49 (9–71)	0.866	0.674	0.861
iSEN neck flexion after surgery	47 (24–69)	47 (22–68)			
iSEN neck extension before surgery	51 (20–79)	49 (20–78)	**0.035**	**0.018**	0.546
iSEN neck extension after surgery	40 (31–51)	34 (30–39)			
iSEN neck lateral flexion before surgery	48 (8–72)	49 (9–71)	0.075	0.058	0.213
iSEN neck lateral flexion after surgery	40 (24–69)	43 (22–68)			
iSEN neck rotation before surgery	60 (24–75)	57 (20–74)	**0.017**	**0.028**	0.704
iSEN neck rotation after surgery	24 (14–52)	26 (14–51)			
Strenght lower trapezius before surgery	13 (7–16)	15 (7–20)	0.499	0.498	0.925
Strenght lower trapezius after surgery	8 (0–14)	10 (0–18)			
Strength middle trapezius before surgery	**15** **(8–19)**	**15** **(10–20)**	**0.028**	**0.012**	0.771
Strength middle trapezius after surgery	8 (0–19)	7 (0–20)			
Strength upper trapezius before surgery	20 (18–23)	22 (17–27)	**0.018**	**0.012**	0.717
Strength upper trapezius after surgery	12 (9–23)	13 (12–24)			
before surgery scapular endurance max	56 (10–143)	63 (17–143)	0.176	0.574	0.141
after surgery scapular endurance max	42 (0–110)	47 (0–110)			
before surgery MCMS	86 (81–94)	87 (76–91)	**0.018**	**0.012**	0.906
after surgery MCMS	73 (53–89)	77 (65–90)			
before surgery SPADI score	0(0–8)	0(0–2)	0.180	0.144	0.950
after surgery SPADI score	6 (0–38)	7 (0–26)			

P1: Wilcoxon Test P2: Wilcoxon Test P3: Repeated Measures Analysis, * CSN: cervical sensitive nerves. Bold numbers means *p* < 0.05.

## Data Availability

The data supporting the findings of this study are available from the corresponding author upon reasonable requests. Owing to institutional policies and ethical restrictions, the dataset is not publicly available. Personal Data Protection Law (Law Number: 6698, Date of Ratification: 24 March 2016, Published in Official Gazette: Date: 7 April 2016, Number: 29677).
